# Crude Oil Sensing using Carbon Nano Structures Synthetized from *Phoenix Dactylifera* L. Cellulose

**DOI:** 10.1038/s41598-019-54417-2

**Published:** 2019-11-28

**Authors:** Chouaib Fethiza Tedjani, Omar Ben Mya, Abdelkrim Rebiai, Abdelhamid Khachkhouche, Abdelhakim Dehbi, Nacer Eddine Méchara

**Affiliations:** 1 Department of Chemistry, Faculty of Exact Sciences, University of El Oued, El Oued, Algeria; 2 Department of Process Engineering & Petrochemistry, Faculty of Technology, University of El Oued, El Oued, Algeria; 3 Laboratory of Valorization of Saharan Resources and its Technologies, University of El Oued, El Oued, Algeria

**Keywords:** Chemistry, Energy science and technology

## Abstract

This study reports on the crude oil-sensing using carbon nano structures (CNSs). A mixture of CNSs was obtained by a simple method of preparation using palm cellulose ash and nitric acid as precursors, the powder was characterized by x-ray diffraction and infrared spectroscopy. The optical density of crude oil from Rhoud El-Baguel area (Southeast of Algeria) studied using UV-Vis spectroscopy, before and after adding an amount of CNSs powder to view the CNSs crude oil sensing and therefore a new method to determine the quality of crude oils and the comparison between them. Results show that CNSs prepared from palm cellulose ash have a good crystallinity and it is formed mainly from carbon nano dots (CNDs) with 4.32 Å in layers spacing and 7.4 Å in crystallite size, indicate that CNSs can be used as an excellent crude oil sensor.

## Introduction

In the petrochemical industry, knowledge of the quality and content of crude oil is a key factor in improving the refining process. Methods of spectroscopy are the most important methods used because they contain important informations regarding the chemical properties of each sample^1^. The compounds in oil, responsible on optical response named: Fluorophores (Table 1), which are compounds that absorbs in the UV-visible range. This condition is fulfilled for conjugated electron systems, such as polyunsaturated molecules and aromatics^2^.

**Table 1:** Some Fluophore found in crude oil^26^.

Fluophore	Chemical structure	Fluophore	Chemical structure
Benzene		Phenol	
p-cresol		dibenzofuran	
Acenaphthene		Carbazole	
Chrysene		Benzo(a)pyrene	
p-methyl anisole		dibenzothiophene	
Phenanthrene		Pyrene	
Porphyrin		Toluene	
m-Xylene		Indole	
Naphthalene		2-Naphthol	
2,3-Benzofuorene		Anthracene	
Perylene			

Nano carbon structures have a wide range of interest because of their use in applications such as energy storage, tribology, electronics, medicine, catalysis and sensors^3–7^. Carbon nano structures (CNSs) are an ultra-small photoluminescent (PL) nanomaterial (<10 nm), It has significant optical properties, disposable surface functions, chemical inactivity, high photoresist, simple and inexpensive methods of preparation, and an abundance of raw materials,…^8^. The preparation of CNSs is a process of mix between two precursor forms, one of which installs the main carbon frame and the other within the structure elements. In this regard, the most prominent synthesis was cellulose ash as a carbon source, while nitrogen acid contains activating molecules^9^.

CNSs are crystals which can act as a sensor by sparkling at the desired wavelength or color. We emphasize that fluorescent organic molecules are often aromatic or contain multiple bonds, which are alternating single and double bonds, responsible for the high-octane number and therefore the quality of petroleum^10^. These molecules contain non-bonding electrons that form a cloud around the molecule and are usually prone to excitement and shine in response to the light energy projected on them^11^.

## Expremental and Methods

### Extraction of Cellulose from Phoenix Dactylifera L. tree

The Phoenix Dactylifera L. leaves were crushed and screened to ensure that the particle size was distributed from 8 meshes to 30 meshes. Leaves were immersed in 5 wt% sodium hydroxide solution at ambient temperature for 12 h. Then they were washed with water for several times and dried in the oven at 80 °C for 24 h. To remove the wax, the debris of leaves were immersed in the solution of methylbenzene and ethyl alcohol (volume ratio of 1:1), and kept boiled for 8 h. The residues were washed with ethyl alcohol several times and then dried in the oven at 80 °C for 24 hours. And to remove lignin, leaves were soaked in hydrogen peroxide (30 vol%) and acetic acid solution (volume ratio of 1:1), and boiled with magnetic stirring at 60 °C for 7 h. Water was used to wash the residue and then filtered until the filter was neutral. The fibers obtained were boiled in 5 wt% of sodium hydroxide solution at 80 °C for 2 h, then, washed with water to neutral and dried in the oven at 80 °C for 24 h. The cellulose fibers from Phoenix Dactylifera L. leaves were obtained^12,13^.

### Synthesis of carbon nanostructures (CNSs)

In order to obtain C-nanostructures, the cellulose extracted previously was carbonized in a muffle furnace directly at 240 °C for 2 h^4^. About 5 g of fine ashes obtained from cellulose furnace and mixed with concentrated nitric acid (60%) and stay in agitation for 24 hours. The mixture was separated by centrifugation at 12,000 rpm for an hour to separate the residue and supernatant. The latter was heated in a vacuum oven at 200 °C^14^.

### Crude oil optic sensing

The oils were excited by ultraviolet rays (300–400 nm) which fluoresce in the visible wavelength range of 400 to 600 nm. The crude oil sample was obtained from Rhoud El-Baguel, close Hassi-Messaoud region, city of Ouargla south eastern of Algeria. To perform optical density measurements, it was required to dilute the sample to obtain a transparent solution to transmit the light. Cyclohexane was chosen as solvent that can optically respond in the range of 350 nm–500 nm, wavelengths used to excite crude oil^15,16^. Six samples of oil diluted in cyclohexane at different concentrations were used for the measurements. Table 2 shows the different concentrations of prepared samples. Absorption spectra of all samples were measured at room temperature. at 350, 400,450 and 500 nm.

**Table 2:** Concentration of oil in cyclohexane.

Sample	Concentration (ml/l)
1	0.5
2	1
3	1.5
4	2
5	2.5
6	3

### Characterization of carbon nanostructures (CNSs) and optic sensing

The type of the carbon Nano structure was analyzed by X-ray powder diffraction (XRD) using a BENCHTOP PROTO AXRD diffractometer in the range 2θ:10–80°(step: 0.1°) and Cu_Kα1_Source (λ = 1,54 Å). Fourier transform infrared spectra were obtained on a SHIMADZU 8400 s (FT-IR) spectrometer whose extent is between 400 and 4000 cm^−1^. UV/visible absorption spectra were recorded with a UV/VIS 6305 spectrophotometer (JENWAY Company).

## Results and Discussion

### Characterization of CNSs by XRD

**Figure 1:**
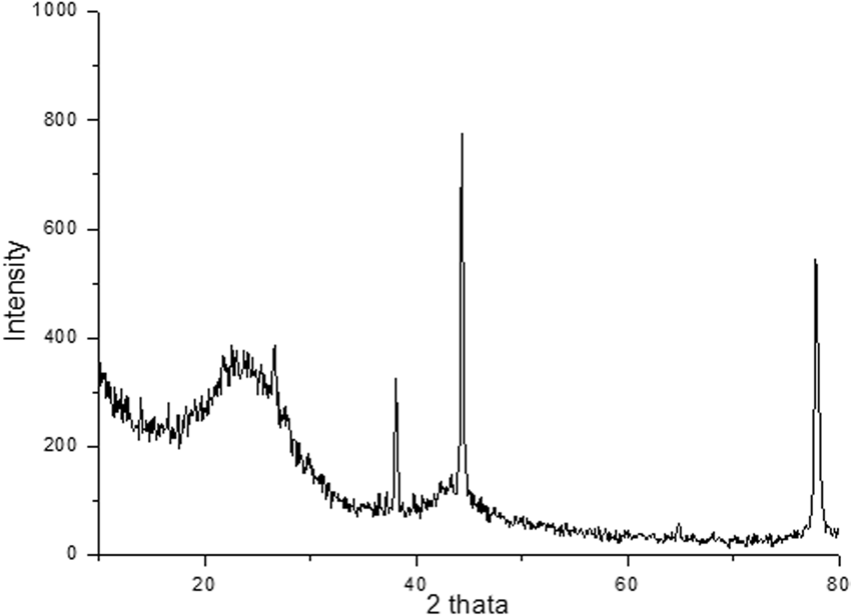
Powder XRD of carbon nano structures sample.

Figure 1 shows the X-ray diffraction pattern of Carbon-Nanostructures (CNSs) produced by one-step thermal carbonization. For carbon nanodots (CNDs), a non-relief reflection band centered on 2θ = 21.68° corresponds the (002) lattice spacing of carbon-based materials with amorphous nature^17^ or shows a shift down; what is indicates an increase in sp2 layer spacing^18^.

Crystal planes and a small broad peak to about 2θ = 44.22° and 77.5° correspond to the set (100) and (110) reflections^19,20^. The spacing between the layers was calculated by applying the Bragg equation and found at approximately 4.23 Å. As long as the average crystallite size, Lc, can be determined using the Scherrer equation:

$${L}_{{\rm{c}}}=\frac{K\lambda }{{\rm{\beta }}\,\cos \,\theta },$$

or:

λ: the wavelength of X-rays (1.54 Å),

β: the width at half height (in radians),

θ: the diffusion angle

and K is the Scherrer constant (0.9)^21^.

The Lc has been estimated at 7.0 Å.

### Infrared spectroscopy FTIR

**Figure 2:**
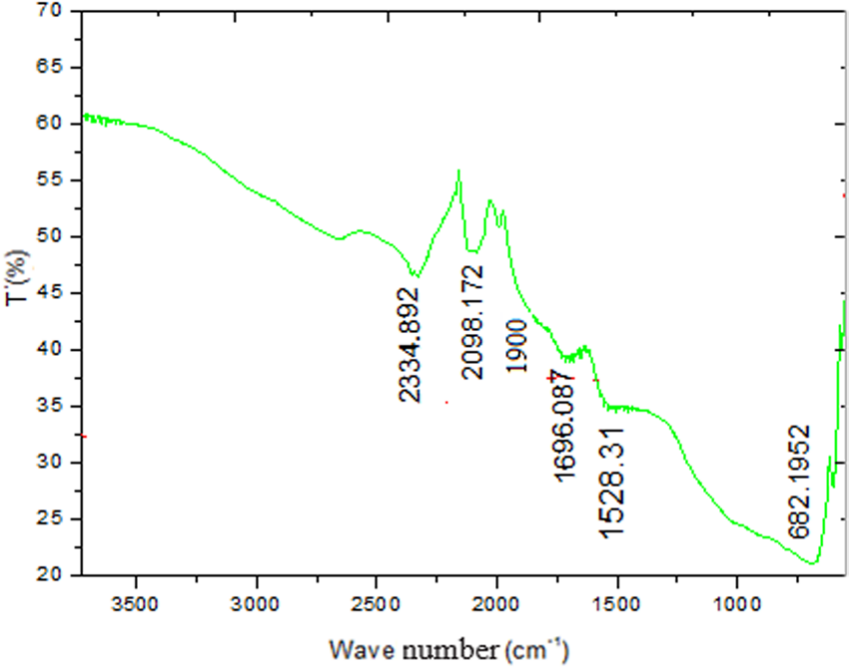
FT IR of CNSs Sample.

As shown in Fig. 2, the existence of carbonyl (C=O) causes the peak of about 1696 cm^−1^. The presence of oxygen-containing carbon structures has been confirmed. The peak at 1528 cm^−1^ can be attributed to the C=C stretching vibrations. The *δ* (C=O) vibration band is found at approximately 680 cm^−1^ ^22,23^. The bands at 1900, 2098.172 and 2334.892 cm^−1^ can been attributed to inorganic ʋ_3_CO_3_, manganese carbonyl stretching frequency and water molecule under strongly hydrogen-bonded conditions^24–26^.

**Figure 3:**
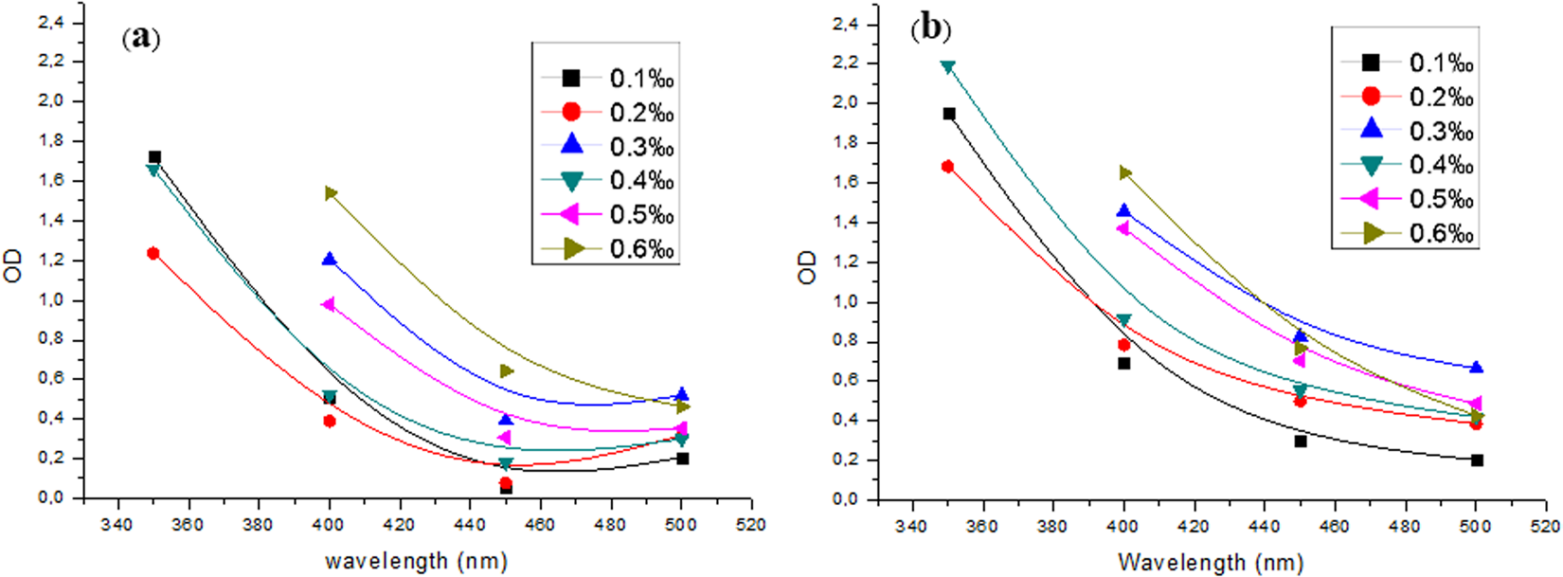
Effect of crude oil sample concentration on optical density at different light wavelengths: (**a**) before and (**b**) after adding CNSs powder.

In Fig. 3a it is possible to observe Rhoud El-baguel crude oil optical properties. It appears that the crude concentration varies proportionally with the optical density (OD) in all the domain of UV-Vis. After adding nano carbon, the concentration 0.4 ml/l shows the best OD (Fig. 3b) throughout the UV-Vis range.

**Figure 4:**
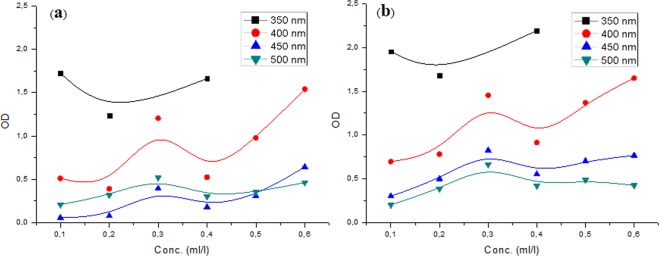
Effect of light wavelengths on optical density of crude oil sample at different concentration: (**a**) before and (**b**) after adding CNSs powder.

The analysis also shows that the samples have a better optical density for a minimum value of wavelength (350 nm) and this for concentrations less than or equal to 0.4 ml/l. While it is less intense for other wavelengths throughout the UV-Vis domain (Fig. 4a). For comparison, the OD increases perfectly according to each concentration and in the whole area at 400 nm (Fig. 4b).

**Figure 5:**
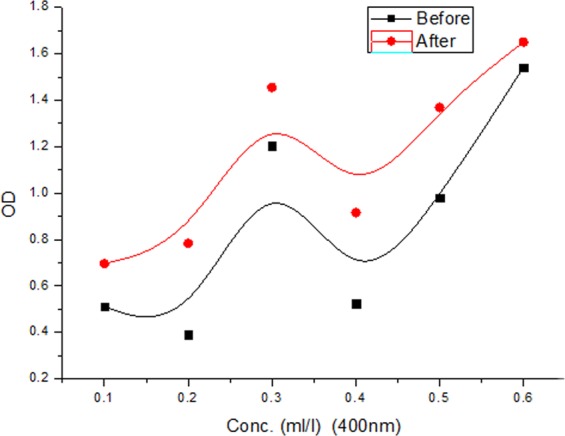
Optical density variation of different oil concentrations at 400 nm before and after adding CNSs powder.

In order to determine clearly the effect of carbon nanostructures on the optical properties of the oil sample, we study the optical density changes in terms of concentration of samples under a constant wave length of 400 nm before and after adding an amount of CNSs powder (Fig. 5).

**Figure 6:**
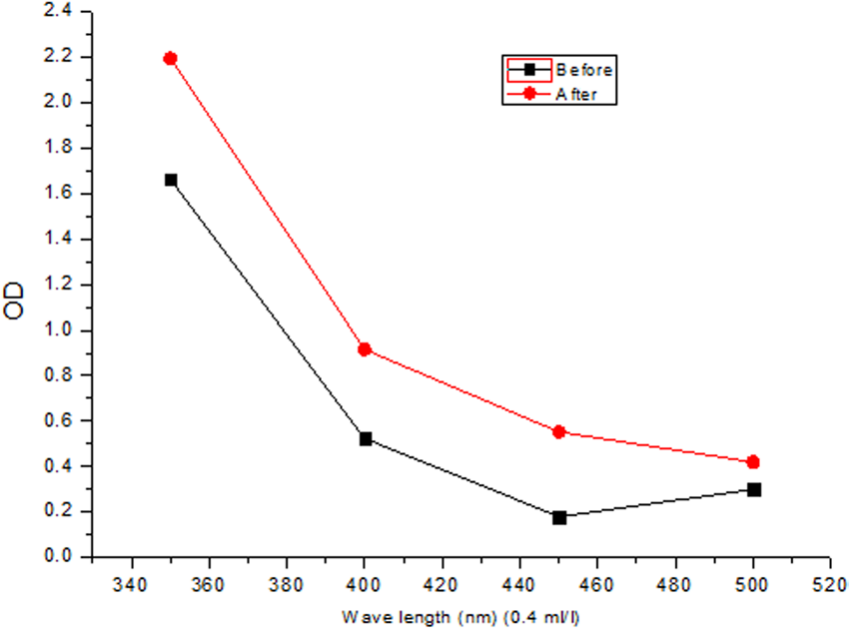
Optical density variation of 0.4 ml/l oil sample at under different light wavelengths before and after adding CNSs powder.

And we study the optical density changes in terms of UV-Vis wavelengths for 0.4 ml/l concentration of sample before and after adding an amount of CNSs powder (Fig. 6). It is clear that the optical density increases strongly by adding nanocarbon.

## Conclusion

Carbon Nanostructures (CNSs) can be synthetized simply with an ash of palm cellulose available locally and maybe used as a very effective tool for sensing and estimating the quality of crude oil and comparing between them.

## References

[1] Steffens J, Landulfo E, Courrol LC, Guardani R (2011). Application of fluorescence to the study of crude petroleum. Journal of fluorescence.

[2] Ellingsen G, Fery-Forgues S (1998). Application de la spectroscopie de fluorescence à l'étude du pétrole: le défi de la complexité. Revue de l’Institut Français du Pétrole.

[3] Taylor TA, Patterson HH (1987). Excitation resolved synchronous fluorescence analysis of aromatic compounds and fuel oil. Analytical chemistry.

[4] Ahmed GHG, Laíño RB, Calzón JAG, García MED (2016). Facile synthesis of water-soluble carbon nano-onions under alkaline conditions. Beilstein journal of nanotechnology.

[5] Wang C (2017). Hierarchical CuCo2O4 nickel-cobalt hydroxides core/shell nanoarchitectures for high-performance hybrid supercapacitors. Science Bulletin.

[6] He, W., *et al* Supercapacitors: Ultrathin and Porous Ni3S2/CoNi2S4 3D‐Network Structure for Superhigh Energy Density Asymmetric Supercapacitors (Adv. Energy Mater. 21/2017). *Advanced Energy Materials* **7**(21), 10.1002/aenm.201770117 (2017).

[7] Wang C, Sun P, Qu G, Yin J, Xu X (2018). Nickel/cobalt based materials for supercapacitors. Chinese Chemical Letters.

[8] Li H, Kang Z, Liu Y, Lee ST (2012). Carbon nanodots: synthesis, properties and applications. Journal of materials chemistry.

[9] Gao F (2017). Rational design of high quality citric acid-derived carbon dots by selecting efficient chemical structure motifs. Carbon.

[10] Kelly JJ, Barlow CH, Jinguji TM, Callis JB (1989). Prediction of gasoline octane numbers from near-infrared spectral features in the range 660-1215 nm. Analytical Chemistry.

[11] Klonoff DC (2012). Overview of fluorescence glucose sensing: a technology with a bright future. Journal of Diabetes Science and Technology.

[12] Ma, N., Liu, D., Liu, Y. & Sui, G. Extraction and characterization of nanocellulose from Xanthoceras Sorbifolia Husks. *Int J Nanosci Nanoeng*, **2**(6), 43–50, http://www.openscienceonline.com/journal/archive2?journalId=731&paperId=2509 (2015).

[13] Manoj B, Ashlin MR, Thomas GC (2018). Tailoring of low grade coal to fluorescent nanocarbon structures and their potential as a glucose sensor. Scientific reports.

[14] Gerrard DL, Maddams WF (1978). Solvent effects in uv absorption spectra. I. Phenol in cyclohexane ethanol mixtures. Spectrochimica Acta Part A: Molecular Spectroscopy.

[15] Pickett LW, Margaret Muntz, McPherson EM (1951). Vacuum Ultraviolet Absorption Spectra of Cyclic Compounds. I. Cyclohexane, Cyclohexene, Cyclopentane, Cyclopentene and Benzene1. Journal of the American Chemical Society.

[16] Bourlinos AB (2012). Gd(III)-doped carbon dots as a dual fluorescent-MRI probe. J Mater Chem..

[17] Zhang HB, Lin GD, Zhou ZH, Dong X, Chen T (2002). Raman spectra of MWCNTs and MWCNT-based H_2_-adsorbing system. Carbon.

[18] Suárez-Garcıa F, Martınez-Alonso A, Díez Tascón JM (2002). Pyrolysis of apple pulp: chemical activation with phosphoric acid. Journal of Analytical and Applied Pyrolysis.

[19] Gupta Vinod, Saleh Tawfik A. (2011). Syntheses of Carbon Nanotube-Metal Oxides Composites; Adsorption and Photo-degradation. Carbon Nanotubes - From Research to Applications.

[20] Smilgies D-M (2009). Scherrer grain-size analysis adapted to grazing-incidence scattering with area detectors. Journal of applied crystallography.

[21] Dhenadhayalan N, Lin KC (2015). Chemically induced fluorescence switching of carbon-dots and its multiple logic gate implementation. Sci Rep..

[22] Moon BJ (2016). Facile and purification-free synthesis of nitrogenated amphiphilic graphitic carbon dots. Chem. Mater..

[23] Glišić S, Nikolić G, Cakić M, Trutić N (2015). Spectroscopic study of copper (II) complexes with carboxymethyl dextran and dextran sulfate. Russian Journal of Physical Chemistry A.

[24] Brangule Agnese, Gross Karlis Agris (2015). Importance of FTIR Spectra Deconvolution for the Analysis of Amorphous Calcium Phosphates. IOP Conference Series: Materials Science and Engineering.

[25] Orgel LE (1962). The Infrared Spectra of Substituted Metal Carbonyls. Inorganic Chemistry.

[26] Furutani Y, Terakita A, Shichida Y, Kandori H (2005). FTIR studies of the photoactivation processes in squid retinochrome. Biochemistry.

